# Clinical and bacteriological characteristics of *Corynebacterium* keratitis

**DOI:** 10.1186/s12348-025-00451-3

**Published:** 2025-01-09

**Authors:** Hidenori Inoue, Koji Toriyama, Shinobu Murakami, Hitoshi Miyamoto, Wakako Ikegawa, Yuki Takezawa, Yuri Sakane, Yuko Hara, Atsushi Shiraishi

**Affiliations:** 1 Department of Ophthalmology, Ehime University Graduate School of Medicine, Shitsukawa, Toon, Ehime 791-0295 Japan; 2 Clinical Laboratory Division, Ehime University Hospital, Shitsukawa, Toon, Ehime 791-0295 Japan; 3 Department of Ophthalmology, Sumitomo Besshi Hospital, Ojicho, Niihama, Ehime 792-8543 Japan

**Keywords:** *Corynebacterium*, Fluoroquinolone resistance, Keratitis, Steroid, Antimicrobial susceptibility

## Abstract

**Purpose:**

*Corynebacterium* species are commensals of human skin and mucous membranes and are recognized as important pathogens in ocular infections. This study investigated the clinical characteristics of *Corynebacterium* keratitis.

**Methods:**

We retrospectively reviewed cases of bacterial keratitis in which *Corynebacterium* species were solely isolated from corneal scraping cultures collected at Ehime University Hospital between January 2010 and February 2024. The clinical findings of *Corynebacterium* keratitis were divided into two types: adherent and infiltrative, which are defined as adhesion to the corneal surface or stromal infiltration, respectively.

**Results:**

Of the 232 culture-positive cases of bacterial keratitis, 23 (9.9%) were positive for *Corynebacterium* species alone. The mean patient age was 60.1 ± 21.0 years, and the cohort included 12 males and 11 females. Adherent type was found in 13 patients (56.5%) and infiltrative type was observed in 10 patients (43.5%). Fluoroquinolone eye drops were used by 14 (60.9%) patients and steroid eye drops by 12 (52.2%). *Corynebacterium macginleyi* was the most commonly identified species (85.7%). 91% of *Corynebacterium* isolates were resistant to fluoroquinolones. All of *C. macginleyi* isolates were fluoroquinolone-resistant, and 93.3% of the isolates were highly resistant (minimal inhibitory concentrations > 32 µg/mL). All cases were treated with frequent antimicrobial eye drops, mainly cephalosporins, and the mean treatment duration was 21.6 days. Although no patient required therapeutic keratoplasty, five adherent types required multiple therapeutic debridements to physically remove the bacteria.

**Conclusions:**

*Corynebacterium* keratitis presented as adherent and infiltrative types of lesions. The main characteristics of the patient included the use of fluoroquinolone and steroid eye drops.

## Background

*Corynebacterium* species are one of the causative organisms of bacterial keratitis [1–3]. They are gram-positive bacilli with a club-shaped morphology, often forming V- or L-shaped “Chinese letter” patterns, and are widely distributed in nature [4]. In the field of ophthalmology, *Corynebacterium* species are endemic to the eyelids and conjunctival sacs, and they are an etiologic agent of conjunctivitis in older adults [5–7]. In recent years, an increasing number of cases of bacterial keratitis due to *Corynebacterium* species have been reported [8–17]. However, only a few reports have examined the clinical features of keratitis caused by *Corynebacterium* species in a larger population. Thus, the details have not been clarified.

In this study, we analyzed the clinical presentation of patients with infectious keratitis in which *Corynebacterium* species were solely isolated from the corneal scrapping cultures, patient backgrounds, identification and in vitro antimicrobial susceptibility of the isolated *Corynebacterium* species, and treatment duration in multiple cases.

## Materials and methods

This study was approved by the Medical Ethics Review Committee of Ehime University Hospital (Approval No. 1503007). We retrospectively reviewed the medical records of patients with bacterial keratitis who visited Ehime University Hospital between January 2010 and February 2024, and in whom only *Corynebacterium* species were solely isolated in the cultures of corneal scrapings. The following data were collected: clinical presentation, sex, age, patient background, bacterial species, antibiotic susceptibility, and treatment.

**Fig. 1:**
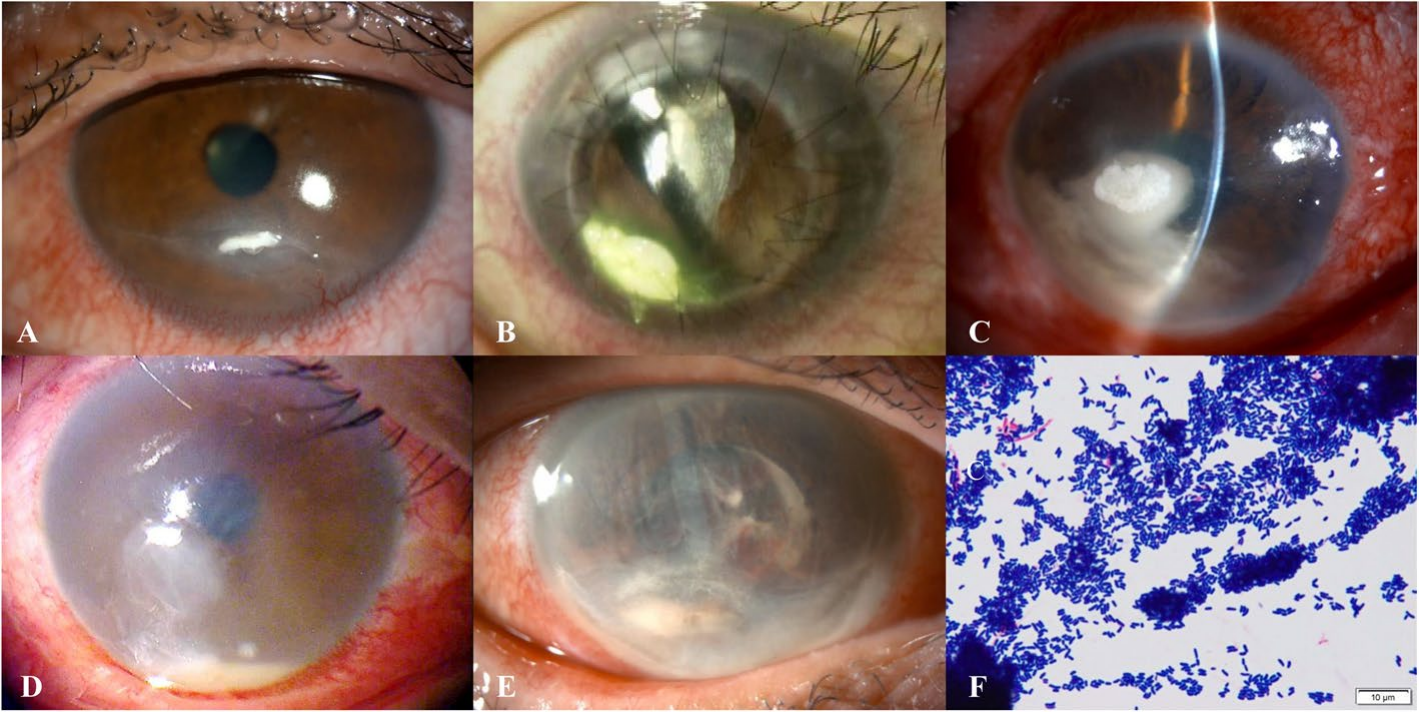
Representative slit-lamp photograph of *Corynebacterium* keratitis. **A**, **B** Adherent type: White plaque adherent to the superficial corneal layer can be visualized. **C** Adherent type with infiltration: White plaque adherent to the superficial corneal layer of the cornea, with infiltration into the corneal stroma can be visualized. **D**, **E** Infiltrative type: Infiltration of the corneal stroma with hypopyon can be visualized. **F** Gram-stained specimen of the corneal surface plaque obtained from a patient with adherent type shows an aggregation of gram-positive rods

Based on lesion appearance, *Corynebacterium* keratitis can be divided into two types: adherent and infiltrative. The adherent type is characterized by clusters of *Corynebacterium* that adhere to the superficial corneal layer, with or without invasion into the corneal stroma (Fig. 1A-C), giving a clinical appearance of various forms of elevated white plaque-like structures. The infiltrative type is characterized by infiltration into the corneal stroma without attachment of the *Corynebacterium* clusters to the superficial corneal layer (Fig. 1D, E). Gram staining of corneal adherents revealed numerous gram-positive rods (Fig. 1F).

The causative organisms were isolated via gram staining microscopy and culture of corneal scrapings on sheep blood agar. The *Corynebacterium* species were identified using matrix-assisted laser desorption ionization time-of-flight mass spectrometry (MALDI-TOF MS) (MALDI Biotyper; Bruker Daltonics, Billerica, MA, USA). Antimicrobial susceptibility testing was performed using a broth microdilution assay. In the present study, the minimal inhibitory concentration (MIC) breakpoints for levofloxacin, cefmenoxime, tobramycin, and azithromycin were established based on the MIC breakpoints for ciprofloxacin, ceftriaxone, gentamicin, and erythromycin, respectively, according to the Clinical and Laboratory Standards Institute M45 criteria [18]. For moxifloxacin and vancomycin, the MIC breakpoints established by the European Committee on Antimicrobial Susceptibility Testing were employed [19]. The MIC breakpoints for gatifloxacin were determined using those established for moxifloxacin.

Data were analyzed using JMP (version 11.2.0; SAS Institute, Cary, NC, USA), using a two-tailed unpaired t-test. A *P*-value of < 0.05 was considered statistically significant.

## Results

### Clinical findings

In the present study, 13 patients (56.5%) had adherent lesions and 10 patients (43.5%) had infiltrative lesions. In almost all the patients, the lesions were located from the paracentral area of the cornea to the inferior part of the cornea. In only one patient, the infiltrative lesion was located in the nasal periphery of the cornea. A hypopyon was observed in six patients (four with adherent and two with infiltrative types). Only one patient with infiltrative type developed corneal perforation.

### Patient backgrounds

During the study period, 232 cases of bacterial keratitis with culture-positive corneal scrapings were identified, of which 23 cases (9.9%) were positive for *Corynebacterium* species alone. This study included a total of 23 cases. The mean age of the 23 patients (12 male and 11 female) was 60.1 ± 21.0 years. Ocular backgrounds were observed in all patients (100.0%) (Table 1). Fluoroquinolone antibacterial eye drops were commonly used (*n* = 14, 60.9%), followed by steroid eye drops (*n* = 12, 52.2%). Seven patients (30.4%) had dry eye, five (21.7%) had previously undergone corneal transplantation, four (17.4%) had a previous history of herpetic keratitis, three (13.0%) had lagophthalmos, and three (13.0%) were contact lens wearers. Systemic backgrounds were identified in seven patients (30.4%). Diabetes mellitus was the most common (*n* = 7, 30.4%), followed by leukemia (*n* = 2, 8.7%). Both patients with leukemia also had diabetes mellitus. The backgrounds for adherent and infiltrative types were almost identical. However, a history of herpetic keratitis and lagophthalmos were only observed in the adherent type.

**Table 1:** Patient backgrounds for *Corynebacterium* Keratitis

	**(*n* = 23), *n* (%)**	**Adherent type (*n* = 13), *n* (%)**	**Infiltrative type (*n* = 10), *n* (%)**
**Ocular backgrounds**	*n* (%)	*n* (%)	*n* (%)
Fluoroquinolone eye drops	14 (60.9)	7 (53.8)	7 (70.0)
Steroid eye drops	12 (52.2)	8 (61.5)	4 (40.0)
Dry eye	7 (30.4)	2 (15.4)	5 (50.0)
Keratoplasty	5 (21.7)	4 (30.8)	1 (10.0)
Herpes keratitis	4 (17.4)	4 (30.8)	
Lagophthalmos	3 (13.0)	3 (23.1)	
Contact lens wear	3 (13.0)	2 (15.4)	1 (10.0)
Others	5 (21.7)		
Total	23 (100.0)		
**Systemic backgrounds**			
Diabetes mellitus	7 (30.4)	3 (23.1)	4 (40.0)
Leukemia	2 (8.7)	1 (7.7)	1 (10.0)
Total	7 (30.4)		

### Bacteriological profile

*Corynebacterium* species were isolated from corneal scrapings in all 23patients. A total of five *Corynebacterium* species were identified by mass spectrometry in 21patients. Several species were identified in two patients. The most frequently identified species was *Corynebacterium macginleyi* (*n* = 18, 85.7%). The other identified species were *Corynebacterium striatum* (*n* = 2, 9.5%), *Corynebacterium propinquum* (*n* = 2, 9.5%), *Corynebacterium pseudodiphtheritium* (*n* = 1, 4.8%), and *Corynebacterium accolens* (*n* = 1, 4.8%).

Of the 26 *Corynebacterium* strains isolated in this study, 22 strains were tested for drug susceptibility (Table 2). The results showed that the resistance rate to fluoroquinolones (levofloxacin, gatifloxacin and moxifloxacin) was 91%. All strains of *C. macginleyi* and *C. striatum* were 100% resistant to all fluoroquinolones. *C. macginleyi* was highly resistant to levofloxacin with MICs of > 32 µg/mL in 14 of 15 strains. Furthermore, 10 of these strains had MICs of ≥ 128 µg/mL. All strains were well susceptible to cefmenoxime and tobramycin and vancomycin (resistance rate, 0%).

**Table 2:** Antimicrobial Susceptibility of *Corynebacterium* Isolates for Keratitis

Antibiotics	*Corynebacterium spp*		*C. macginleyi*		*C. propinquum*		*C. striatum*		others	
	**(*n* = 22)**		**(*n* = 15)**		**(*n* = 2)**		**(*n* = 2)**		**(*n* = 3)**	
	**MIC (μg/mL) range**	**% R**	**MIC (μg/mL) range**	**% R**	**MIC (μg/mL) range**	**% R**	**MIC (μg/mL) range**	**% R**	**MIC (μg/mL) range**	**% R**
Levofloxacin	0.5- > 128	91	32- > 128	100	1–32	50	8–64	100	0.5–64	67
Gatifloxacin	≦0.5- > 32	91	8- > 32	100	≦0.5–2	50	4–16	100	≦0.5–16	67
Moxifloxacin	≦0.5- > 32	91	8- > 32	100	≦0.5–2	50	4–16	100	≦0.5–16	67
Cefmenoxime	≦2	0	≦2	0	≦2	0	≦2	0	≦2	0
Tobramycin	≦2–4	0	≦2	0	≦2	0	≦2–4	0	≦2	0
Azithromycin	≦0.25- > 16	45	≦0.25–16	27	≦0.25- > 16	50	> 16	100	> 16	100
Vancomycin	≦1	0	≦1	0	≦1	0	≦1	0	≦1	0

*Abbreviations*: *MIC* Minimum inhibitory concentration, *%R* Resistance rate

### Treatment

All patients were treated with frequent topical antimicrobial eye drops (Table 3). In some patients, systemic antimicrobials were administered and frequent corneal scrapings were obtained. Cephalosporins (cefmenoxime) were the most frequently used topical antibacterial agents (*n* = 21, 91.3%), followed by fluoroquinolones (levofloxacin, gatifloxacin, and moxifloxacin; *n* = 9, 39. 1%), aminoglycosides (tobramycin; *n* = 6, 26.1%), and glycopeptides (vancomycin; *n* = 2, 8.7%). Eight patients were treated with cephalosporins alone, and 15 patients were treated with a combination of two antimicrobial agents. The most common combination was a cephalosporin/fluoroquinolone (*n* = 9), followed by a cephalosporin/aminoglycoside (*n* = 4). The combination of topical and systemic antimicrobials was used in only one patient. Macrolides (clarithromycin) were administered systemically in this patient.

**Table 3:** Management Procedures for *Corynebacterium* Keratitis

Topical Management	*n* (%)
Cephalosporin	
Cefmenoxime	21 (91.3)
Fluoroquinolone	
Levofloxacin	3 (13.0)
Gatifloxacin	3 (13.0)
Moxifloxacin	3 (13.0)
Total	9 (39.1)
Aminoglycoside	
Tobramycin	6 (26.1)
Glycopeptide	
Vancomycin	2 (8.7)

In the present study, some patients with adherent type required frequent corneal scrapings. All patients with infiltrative type required only one corneal scraping at the time of diagnosis. Five of the 13 patients with adherent type required frequent corneal scrapings of the lesion to remove the clusters of *Corynebacterium* species. The mean number of scrapings required for patients with adherent type was 2.1 (range, 1–6).

Treatment duration was defined as the period during which frequent antimicrobial eye drops (≥ 6 times per day) were required. The mean treatment duration was 21.6 days (range, 5–62 days) in 19 patients in whom the treatment duration could be identified. The mean treatment duration was 25.5 days (range, 5–62 days) for adherent type and 16.1 days (range, 7–56 days) for the infiltrative type. Although the treatment duration of adherent type was longer than that of infiltrative type, it was not statistically significant (*p* = 0.30, two-tailed unpaired t-test). Based on the use of steroid eye drops prior to disease onset, the treatment duration in the nine patients who used steroid eye drops averaged 25.0 days (range, 7–56 days), while the treatment duration the 10 patients who did not use steroid eye drops averaged 18.4 days (range, 5–62 days). There was no statistically significant difference between the two groups (*p* = 0.46, two-tailed unpaired t-test). No patients required therapeutic keratoplasty.

## Discussion

*Corynebacterium* species are endemic to the conjunctival sac and are not highly pathogenic [20–22]. Therefore, when *Corynebacterium* species are detected in ocular infections, it is important to determine whether they are pathogenic or not. In this study, we focused on cases with a strong suspicion of infectious keratitis based on clinical findings, in which *Corynebacterium* species were solely identified from corneal scrapings and therefore considered pathogenic. To the best of our knowledge, this is the first review of *Corynebacterium* keratitis in Japan and the first study that has examined the shape of lesions in multiple patients.

In this study, the clinical findings of keratitis caused by *Corynebacterium* species were classified into two types: adherent and infiltrative (Fig. 1). Gram staining of the adherent material revealed numerous gram-negative rods. This suggests that *Corynebacterium* species may adhere to the corneal surface layer, formed a bacterial colony, and produced a biofilm. *Corynebacterium* species adhere to biomaterials such as catheters and corneal sutures and form biofilms [8, 23]. On the other hand, Mihara et al. reported a case in which *Corynebacterium* species adhered to the scleral cornea and formed a biofilm and colonies [24], suggesting that *Corynebacterium* species may form colonies on the ocular surface without biomaterial.

The major risk factors for *Corynebacterium* keratitis are reportedly ocular surface disease [13, 17] and contact lens use [15]. In this study, ocular surface diseases such as dry eye and lagophthalmos and contact lens use were also recognized as risk factors. Soni et al. also reported that the use of steroid eye drops was an important risk factor [17]. Similarly, in this study, the use of steroid eye drops was observed in more than half of the patients. Furthermore, 30.4% of our patients had diabetes mellitus. *Corynebacterium* species are attenuated bacteria [20]. Thus, a deficiency in ocular and systemic immunity may be important triggers for *Corynebacterium* keratitis. Eguchi et al. reported that *C. macginleyi*, clinical ophthalmic isolates, is highly resistant to fluoroquinolones [25]. Suzuki et al. reported two cases of keratitis due to *C. macjinleyi* that were induced by long-term use of fluoroquinolone eye drops after corneal transplantation [8]. In this study, the predominance of fluoroquinolone-resistant *Corynebacterium* species on the ocular surface due to the selective pressure by the use of fluoroquinolone eye drops could have contributed to the development of *Corynebacterium* keratitis.

In our study, all patients with a history of herpetic keratitis or lagophthalmos exhibited an adherent lesion. In lagophthalmos and decreased corneal sensation due to herpetic keratitis, blinking decreases, which may facilitate the adhesion of *Corynebacterium* to the corneal surface and contribute to the formation of the adherent lesion. Further studies are required to determine the triggers that contribute to lesion formation.

In our study, MALDI-TOF MS revealed that 85.7% of the causative organisms were *C. macginleyi*. In a report by Hoshi et al. 84% of *Corynebacterium* species isolated from the conjunctival sacs were *C. macgileyi* [26]. Sagerfors et al. reported 29 cases of keratitis in which *C. macginleyi* was identified [15], and Suzuki et al. reported two cases of keratitis caused by *C. macginleyi* [8]. Based on the results of our study and those of previous studies, *C. macginleyi* may be a major *Corynebacterium* species that is endemic on the ocular surface and a causative agent of keratitis.

In this study, the drug susceptibility of the isolated *Corynebacterium* species was high. However, the resistance rate to any of the fluoroquinolones was 91%, with *C. macginleyi* strains demonstrating a 100% resistance rate. Reports of *Corynebacterium* keratitis from other countries have stated a resistance rate of 0% to 58.3% to fluoroquinolones among the *Corynebacterium* species, which is much lower than that in our study [13, 15, 17]. *Corynebacterium* species do not possess topoisomerase IV, and only DNA gyrase mutations cause resistance, making *Corynebacterium* species more likely to become resistant to fluoroquinolones [27, 28]. Therefore, the overuse of fluoroquinolones in Japan may have resulted in more *Corynebacterium* species becoming resistant to this antimicrobial. In the present study, *C. macginleyi* that was highly resistant to levofloxacin (MIC > 32 µg/mL) was detected in 14 of 15 isolates (93.3%). Furthermore, 10 of these isolates had an MIC ≥ 128 µg/mL. Eguchi et al. found that a double mutation of amino acids 83-S and 87-D of GyrA in the DNA gyrase of *C. macginleyi* induces highly resistance to fluoroquinolones [25]. It is possible that the strain identified in this study also had a double mutation of the GyrA amino acid.

In the present study, topical cefmenoximes were widely used. Cefmenoxime is a third-generation cephalosporin with a broad spectrum of activity against both gram-positive and gram-negative bacteria. It is particularly effective in ocular infections because of its good penetration into ocular tissues and low resistance rates among ocular pathogens. However, cefmenoxime is not widely available in many regions. Thus, potential alternatives, such as cefazolin (a first-generation cephalosporin) and vancomycin (a glycopeptide), which are also effective against gram-positive bacteria including *Corynebacterium* species, should be considered. In previous reports, *Corynebacterium* keratitis has been treated with topical cephalosporins [8–10, 12, 29]. The isolates in this study showed good susceptibility to cephalosporins, with a resistance rate of 0%. Considering these results, in cases of infectious keratitis, when *Corynebacterium* species are suspected as the causative pathogen based on patient background, clinical findings, and gram staining microscopy and culture of corneal scrapings, fluoroquinolone treatment should be avoided, and cephalosporins should be used instead.

None of our cases required therapeutic keratoplasty. In contrast, previous reports showed that 8.3% to 18.2% of patients required keratoplasty [13, 17]. It is possible that frequent use of topical cephalosporins from the beginning of treatment in almost our cases may have been effective. Some of our patients with adherent type required frequent corneal scrapings. *Corynebacterium* species have been reported to generate biofilms on the ocular surface and form plaques, which are clusters of bacteria [8, 24]. Because bacteria in biofilms are resistant to antimicrobial agents [30] and can evade the host immune response [31], adherent lesions, which are clusters of biofilm-covered bacteria, may be difficult to treat with conservative therapy. Thus, physical removal of the bacteria may be useful for treatment.

Our study has several limitations. First, it was a relatively small, retrospective, single-center study with a limited sample size. More extensive studies are required to characterize the clinical features in detail. Second, the mechanisms underlying the development of the two types of keratitis remain unclear. In vivo models of *Corynebacterium* keratitis need to be established to study the factors involved in the pathogenesis of keratitis.

## Conclusions

*Corynebacterium* keratitis presents as an adherent or infiltrative type. The use of fluoroquinolone antimicrobial eye drops and steroid eye drops could be important patient backgrounds for *Corynebacterium* keratitis. Because most of the *Corynebacterium* species isolated from keratitis are resistant to fluoroquinolones, we should be careful in choosing antimicrobials for the treatment.

## Data Availability

No datasets were generated or analysed during the current study.

## Abbreviations

MALDI-TOF MS: Matrix-Assisted Laser Desorption Ionization Time-Of-Flight Mass Spectrometry
MIC: Minimal Inhibitory Concentration
